# Sharing Digital Health Educational Resources in a One-Stop Shop Portal: Tutorial on the Catalog and Index of Digital Health Teaching Resources (CIDHR) Semantic Search Engine

**DOI:** 10.2196/48393

**Published:** 2024-03-04

**Authors:** Julien Grosjean, Arriel Benis, Jean-Charles Dufour, Émeline Lejeune, Flavien Disson, Badisse Dahamna, Hélène Cieslik, Romain Léguillon, Matthieu Faure, Frank Dufour, Pascal Staccini, Stéfan Jacques Darmoni

**Affiliations:** 1 Department of Digital Health Rouen University Hospital Rouen France; 2 LIMICS, INSERM U1142 Sorbonne Université Paris France; 3 Department of Digital Medical Technologies Holon Institute of Technology Holon Israel; 4 European Federation for Medical Informatics Le Mont-sur-Lausanne Switzerland; 5 SESSTIM Aix Marseille Univ APHM, INSERM, IRD, Hop Timone, BioSTIC Marseille France; 6 Department of Pharmacy Rouen University Hospital Rouen France; 7 Délégation du Numérique en Santé Paris France; 8 RETINES Université de Nice Côté d'Azur Nice France

**Keywords:** digital health, medical informatics, medical education, search engine, knowledge management, semantic web, language, teaching, vocabulary, controlled, students, educational personnel, French, curriculum

## Abstract

**Background:**

Access to reliable and accurate digital health web-based resources is crucial. However, the lack of dedicated search engines for non-English languages, such as French, is a significant obstacle in this field. Thus, we developed and implemented a multilingual, multiterminology semantic search engine called *Catalog and Index of Digital Health Teaching Resources* (CIDHR). CIDHR is freely accessible to everyone, with a focus on French-speaking resources. CIDHR has been initiated to provide validated, high-quality content tailored to the specific needs of each user profile, be it students or professionals.

**Objective:**

This study’s primary aim in developing and implementing the CIDHR is to improve knowledge sharing and spreading in digital health and health informatics and expand the health-related educational community, primarily French speaking but also in other languages. We intend to support the continuous development of initial (ie, bachelor level), advanced (ie, master and doctoral levels), and continuing training (ie, professionals and postgraduate levels) in digital health for health and social work fields. The main objective is to describe the development and implementation of CIDHR. The hypothesis guiding this research is that controlled vocabularies dedicated to medical informatics and digital health, such as the Medical Informatics Multilingual Ontology (MIMO) and the concepts structuring the French National Referential on Digital Health (FNRDH), to index digital health teaching and learning resources, are effectively increasing the availability and accessibility of these resources to medical students and other health care professionals.

**Methods:**

First, resource identification is processed by medical librarians from websites and scientific sources preselected and validated by domain experts and surveyed every week. Then, based on MIMO and FNRDH, the educational resources are indexed for each related knowledge domain. The same resources are also tagged with relevant academic and professional experience levels. Afterward, the indexed resources are shared with the digital health teaching and learning community. The last step consists of assessing CIDHR by obtaining informal feedback from users.

**Results:**

Resource identification and evaluation processes were executed by a dedicated team of medical librarians, aiming to collect and curate an extensive collection of digital health teaching and learning resources. The resources that successfully passed the evaluation process were promptly included in CIDHR. These resources were diligently indexed (with MIMO and FNRDH) and tagged for the study field and degree level. By October 2023, a total of 371 indexed resources were available on a dedicated portal.

**Conclusions:**

CIDHR is a multilingual digital health education semantic search engine and platform that aims to increase the accessibility of educational resources to the broader health care–related community. It focuses on making resources “findable,” “accessible,” “interoperable,” and “reusable” by using a one-stop shop portal approach. CIDHR has and will have an essential role in increasing digital health literacy.

## Introduction

### Background

Medicine, health care, and wellness will become increasingly digitized. Thus, digital technologies are more than ever taking a pivotal position in clinical practice, making it crucial to educate future professionals to efficiently grasp digital health and health informatics [1,2]. The World Health Organization views digital health as “a broad umbrella term encompassing eHealth, mHealth, as well as emerging areas, such as the use of advanced computing sciences in big data, genomics, and artificial intelligence.” The World Health Organization affirmed that to strengthen health systems using digital health technologies, finding ways to build capacity and creating a digitally capable health workforce should be key objectives [3,4].

The integration of digital technologies has brought about significant changes in the realm of health professions education. Our research identified various digital education–related inquiries, culminating in a comprehensive and diverse research agenda. We proposed a conceptual framework to assist educators and researchers in developing, designing, and studying digital education. However, we acknowledge the need for further data from lower- and middle-income countries [5].

In 2022, the Delegation of Digital Health of the French Ministry of Health and the French National Research Agency published an open call for projects to support the development of digital health teaching and learning technologies, in French, and dedicated to the community of French health–related professions students and practitioners [6]. These include medicine; dental medicine; pharmacy; midwifery; nursing; physiotherapy; ergotherapy; and, more broadly, any related field such as social work, health administration, and biomedical engineering. By 2027, this heterogeneous community, which includes postgraduates and continuous learners, will reach 210,000 members trained simultaneously in France.

Thus, the association of the departments of digital health (DDHs) of the University of Rouen Normandy (URN) and Côte d’Azur University (CAU) is developing and implementing the SaNuRN (*Santé Numérique Rouen Nice*) [7], a 5-year project started in September 2022 and granted with €3,951,200 (US $4,163,775) for a total cost of €6,891,923 (US $7,262,708), in the context of the said open call (grant #ANR_22-CMAS-0014) having an overall budget of €71 million (US $77.6 million) dedicated by the government to digital health education.

From an educational perspective, SaNuRN is currently based on existing pedagogical resources developed by the DDHs of URN and CAU. In addition, a large part of these resources follows the concepts structuring the French National Referential on Digital Health (FNRDH) [8] that provides French higher education institutions educating health-related professionals with a guideline to support teaching in digital health. Thus, students and lecturers from URN, CAU, and other higher education institutions and professionals have free and unrestricted access to the *Catalog and Index of Digital Health Teaching Resources* (CIDHR) as a platform providing structured and validated information contributing to the body of knowledge necessary to master the field [9].

For example, since 1993, the URN DDH has been developing CISMeF (*Catalogue et Index des Sites Médicaux en langue Française*; in English, Catalog and Index of Medical Sites in French Language), a catalog of French-speaking health resources currently containing 128,689 inputs, including 9409 teaching resources. Moreover, since 1999, with the foundation of the French Medical Virtual University [10], all these teaching resources have been freely available in open access [11,12].

Dealing with teaching material in digital health for academic purposes is challenging because of the availability of many resources. However, the French-speaking material is globally limited compared with the one available in English. Therefore, we are developing the CIDHR [9].

In contrast to other educational platforms that mainly cater to English speakers and require payment, such as the Healthcare Leadership Academy [13], various platforms supported by the UK National Health Service [14], or the IMD Health cloud-based platform [15], CIDHR plays an important role in freely engaging French-speaking students and the health care practitioners community in digital health teaching and learning.

One of the primary reasons for emphasizing the need for a French-speaking knowledge catalog in the digital health domain, such as CIDHR, is to bridge the language gap. Although English is a dominant language in scientific literature and teaching platforms, it excludes a substantial portion of the global population, particularly those more comfortable with other languages and, more particularly, French in this specific case. Thus, this language barrier can hinder the dissemination of critical information and knowledge transfer in digital health education and the development of a dedicated platform in French (which can comprise resources in other languages) [16-19].

From an informatics perspective, SaNuRN is based on semantic technologies. Since 2000, the DDH of URN has been developing and maintaining a semantic search engine (Doc’CISMeF) that was developed using primarily the Medical Subject Headings (MeSH) thesaurus [20] to manage the CISMeF resources. Starting in 2010, a multiterminology and multilingual approach is being continuously developed and used to allow any CISMeF resource to be indexed by more than 1 health terminology and by more than 1 language, although the MeSH thesaurus remains the pivotal terminology and, for CISMeF, the French and the English are the 2 pivotal languages [21,22].

**Figure 1:**
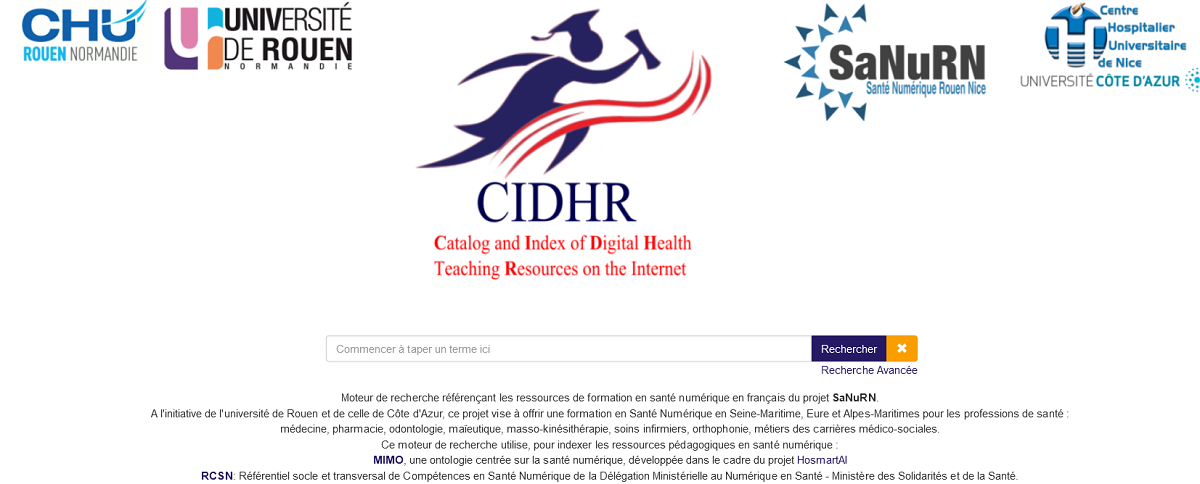
The Catalog and Index of Digital Health Teaching Resources (CIDHR) portal in French.

As a natural evolution with the goal to share as much as possible the open access resources, and within the SaNuRN framework, starting in 2022, we have been developing and implementing a multilingual multiterminology semantic search engine CIDHR. We focus on continuously expanding CIDHR to fit the goal of the SaNuRN project and facilitating the daily teaching and learning practice in medical education by offering easy-to-use indexation and retrieval processes of any educational resource in digital health mainly toward not only French speakers but also toward others; the portal is available among other languages in English, German, Spanish, Greek, Croatian, Chinese (Mandarin), and Finish (Figure 1 [9]).

### Aim, Objective, and Hypothesis

Our main aim in developing and implementing CIDHR, as a multilingual multiterminology semantic search engine, is to enhance knowledge sharing and spreading in digital health and health informatics and to expand the health-related educational community, primarily French speaking but also in other languages [23]. In particular, we aim to support the continuous development of initial (ie, bachelor level), advanced (ie, master and doctoral levels), and continuing training (ie, professionals and postgraduate levels) in digital health for health and social work fields.

Our main objective is to describe the development and implementation of the semantic search engine CIDHR in SaNuRN as a way to foster digital health education and continuous training in France. The hypothesis that guided this research is that controlled vocabularies dedicated to medical informatics and digital health, such as the Medical Informatics Multilingual Ontology (MIMO) [24,25] and the concepts structuring the FNRDH [8], to index digital health teaching and learning resources, are effectively increasing the availability and accessibility of these resources to medical students and other health care professionals.

## Methods

### Highlights

CIDHR is a part of the SaNuRN project. To better understand how we are developing and implementing CIDHR as a catalog of indexed digital health resources, we present the methodological steps in this process in the next lines. First, resource identification is processed by medical librarians; then, based on controlled vocabularies (an ontology and a competency referential organized as a taxonomy), the teaching and learning resources are indexed for each related knowledge domain. In the third step, the same resources are tagged with relevant academic and professional experience levels. The fourth step consists of sharing the indexed resources with the digital health teaching and learning community (with some focus on the French-speaking community). The last step consists of assessing CIDHR by obtaining informal feedback from users.

### Resources Identification

To identify new or updated digital health teaching and learning resources, a group of 3 librarians from URN DDH is working on a continuous information watch, according to an internally developed and validated process comprising the steps and actions.

Thus, the librarians search proprietarily on a predefined list of academic websites of Schools of Health Sciences (eg, Medicine, Dental Medicine, Pharmacy, Nursing, Rehabilitation), National Agencies (eg, the French Ministry of Health [26]; the French National Authority for Health—*La Haute Autorité de Santé* [27]; the French national agency for medicines and health products safety—*Agence Nationale de sécurité du médicament*; and the French Agency for Food, Environmental and Occupational Health & Safety—*Agence Nationale de Sécurité Sanitaire de l’Alimentation, de l’Environnement et du Travail*); and other organizations involved in digital health education such as universities in France and around the world. They are also using search engine alerts, allowing reception of emails with potentially interesting content detected by their algorithms.

Moreover, the librarians monitor social media platforms, such as X (formerly known as Twitter), LinkedIn, or Facebook, by following and screening digital health–related accounts and groups sharing potentially relevant educational supports in digital health and health care informatics. The same search is performed by reading newsletters from professional organizations and academic institutions.

Furthermore, direct contacts with librarians and professional networks in digital health, particularly in the educational field, are used to obtain early updates about new and updated resources before their publishing over the web.

Resource identification also comprises the users’ engagement with CIDHR as a platform, which can share their comments with the whole team (not only the librarians) and suggest additional resources.

Therefore, by using a variety of identification approaches, the librarians involved in CIDHR can propose to the digital health experts of the SaNuRN project a wide range of digital health educational resources to integrate. It is critical to remember that the resources identified are multilingual (although mostly in French because of the SaNuRN grant requirements).

Librarians evaluate each potential resource against the following three criteria:

1. Is the resource a digital health or health informatics education–related one? The resource should be designed to teach users or to support their teaching (depending on whether the user is a student or a lecturer).
2. Is the resource accurate and up-to-date? The resource should be based on current research and best practices.
3. Is the resource accessible? The resource should be available to many users, including those with disabilities.

If a resource meets all 3 criteria, the resource is added to the SaNuRN or CIDHR repository for tagging and indexing. If a potential resource fails the evaluation, it is excluded, at least temporarily, until the librarians recheck the resource and its positive compliance with the evaluation criteria.

### Resources Indexation

For indexing the identified educational resources, CIDHR uses 2 knowledge organization systems (KOSs).

The first is the MIMO, which comprised 3645 concepts in 33 languages as of September 2023 [23-25]. An ontology formally represents a set of concepts within a domain and the relationships between these concepts.

The second KOS was the FNRDH created in 2021. Specifically, FNRDH describes 29 different competencies and 70 different abilities. FNRDH has a 3-level hierarchy. The first relies on 5 main competencies (health data, communication in health, digital tools in health, telehealth, and cybersecurity). The second level relies on 25 subcompetencies (eg, characterizing and managing nominative data, applying [European] regulation [in particular General Data Protection Regulation]), and the last level describes 70 different abilities (eg, understanding the life cycle of the digital health data) [8].

As a side note, MIMO and FNRDH are freely available through the Health Terminology/Ontology Portal [28], also developed by URN DDH over the past 20 years [29,30]. These 2 KOSs are used at an automated stage wherein the resources are preindexed based on keyword identification and then through a librarian indexation validation stage or manual indexation if the automated process is invalid.

Moreover, CIDHR is built around 2 sets of metadata (SoM): the Learning Object Metadata (LOM) set [31] and the Dublin Core Metadata Terms (DCMI-MT) set [32]. LOM is a standard for describing digital learning resources. It provides a set of metadata elements that can be used to describe the characteristics of a learning resource such as its title, description, educational objectives, and technical requirements. DCMI-MT is a simple metadata schema that can describe various digital resources. It provides a set of 15 core metadata elements, including title, creator, and subject. Both SoM are transparent for the final user and allow efficient management of the overall available data related to a selected education resource for being included in CIDHR. These SoM are autocompleted when metadata are available with a resource (ie, a website) and are then validated by a medical librarian. If the automated process fails, the librarian handles this task.

Using 2 KOSs and 2 SoM allows a flexible and comprehensive organization of CIDHR. First, the combination of the KOSs, MIMO as an ontology, and FNRDH as a referential provides a structured way to describe the concepts and skills covered by the teaching resources. Second, the SoM provide a way to describe the characteristics of the teaching and learning resources themselves. Combining KOSs and SoM makes it easy for users to find the appropriate educational resources.

For example, a user (eg, a medical student) interested in learning about the use of artificial intelligence in digital health can use CIDHR to find learning resources that are indexed with the following MIMO concepts: “artificial intelligence,” “digital health,” “machine learning,” and “data mining”; or the same user can find resources indexed with the following FNRDH skill: “use of artificial intelligence in digital health.” Accordingly, CIDHR provides a list of relevant educational resources.

Using KOSs and metadata sets is a common practice in digital learning to organize and represent digital learning resources in a flexible, comprehensive, and user-friendly manner.

### Resources Tagging and Integration to the Curricula

Resource indexation is a critical stage of the CIDHR knowledge management process and a pivotal component of the overall SaNuRN project. However, the main aim is to use CIDHR as a support for digital health learning and teaching in integrating the medical and health-related undergraduate, postgraduate, and life continuing education curriculum. It is also important to suggest the right resources to the specific end user (ie, student according to his degree and field of study and lecturer according to his students and his field of teaching). Thus, LOM and its instantiation in France, known as SupLomFr [33], and DCMI-MT were previously used in CISMeF that we have introduced above [11].

Thus, the 2 leading metadata are of utmost importance to help health-related students and lecturers find the right educational resources at the right time.

The first metadata is the “field of study” (ie, initial long-path education [>5 years]: medicine [Doctor of Medicine], dental surgery [Doctor of Dental Surgery], pharmacy [PharmD], and midwifery [State Diploma of Midwifery]; initial short-path education [until 5 years]: nursing [registered nurse], physiotherapy [State Diploma of Physiotherapist], and occupational therapy [State Diploma of Occupational Therapist]; and social work [State Diploma of Social Worker]).

The second metadata is the “degree level” (bachelor, master, doctorate, or residency in medicine, dental surgery, and pharmacy). It is important to point out that the graduates of an initial short-path education can continue their education in their fields at the postgraduate levels (master and doctorate degrees and lifelong continuing education).

Therefore, for any query performed on CIDHR, the end user may select and save these 2 metadata, “field of study” and “degree level” (eg, “Nursing” AND “Master Degree”; “Medicine” AND “Residency”). The so-called “training matrix” is generated to provide each combination of learners with a set of resources relevant to their profile. This set of educational resources is defined by consensus by the SaNuRN pedagogical team to be the most exhaustive. The “training matrix” is periodically updated according to the introduction of new resources or updates.

Moreover, any kind of teaching resource is cataloged in CIDHR, thanks to an extensive resources type hierarchy created for CISMeF based on a conceptual extension of the MeSH publication type [20,34]. This resource-type hierarchy has been used fruitfully for more than 20 years by users (health students, academics, and professionals) of the CISMeF platform searching for clinical-focused resources.

**Figure 2:**
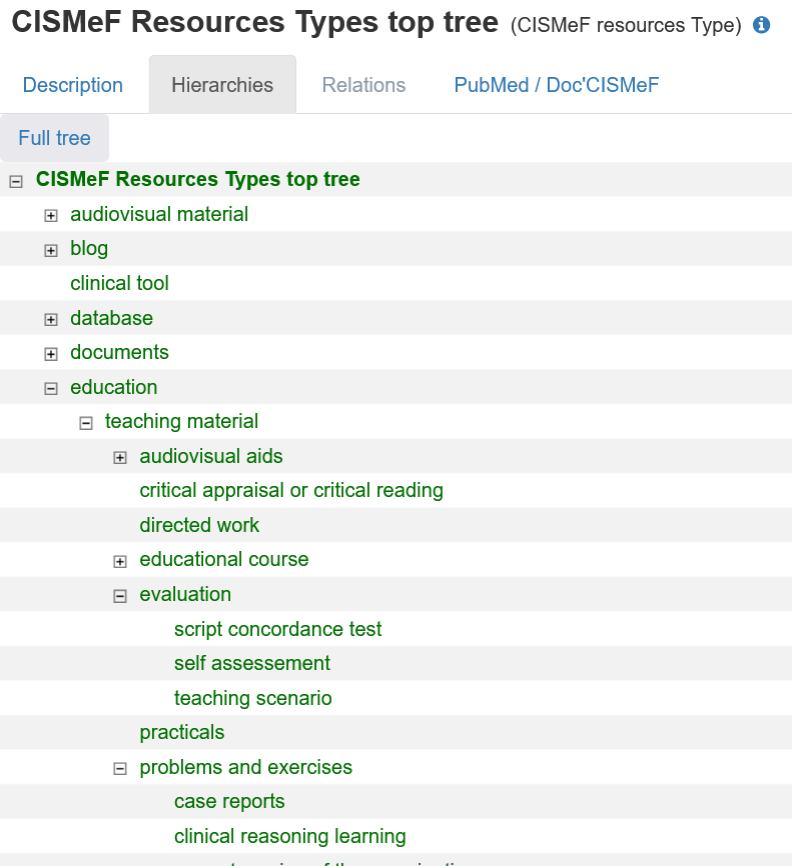
List (sample) of resource types for teaching resources in the Health Terminology/Ontology Portal. CISMeF: Catalogue et Index des Sites Médicaux en langue Française (Catalog and Index of Medical Sites in French Language).

The following teaching resources are cataloged by tagging each one based on the following resource-type hierarchy (Figure 2 [28,35,36]): a “classical” teaching resource supporting a face-to-face course delivered with a series of slides (resource type: teaching material); evaluation of knowledge, such as multiple-choice question; and evaluation of competence, such as Objective Structured Clinical Examination or Script Concordance Test. These last 2 innovative approaches used as competency evaluation tools have been proposed for the nursing curriculum [37]; their use will be extended to other fields in CIDHR.

These combinations of the metadata tags “field of study” and “degree level” with the “resource type” tag as filters allow delivery to the user more or fewer indexed resources relevant to the knowledge fields submitted in the query to CIDHR depending on the filters selection submitted with the query.

### User Experience Assessment

To assess the reception of CIDHR among users, we conducted an informal assessment including the following steps. First, a group of users consisting of both students (health students in their first year: 10/150, 6.7%) and the 5 teaching staff of digital health (JG, AB, PS, RL, and SJD) from diverse educational backgrounds and institutions was recruited. Then, immediately after the first set of lessons, the student participants were given access to CIDHR and encouraged to explore its features, search for digital health resources, and interact with the platform over a few days. Afterward, each user involved was invited to share, during a short interview, their feedback about their (1) perception of CIDHR’s user-friendliness and “easily navigable” capabilities; (2) comments on content quality comprehensiveness and the ongoing expansion; and (3) perception of CIDHR as a one-stop shop for freely and unrestricted accessible, primarily available digital health resources in their academic (ie, learning, teaching, and research) and professional activities. The last component of the feedback collection consisted of obtaining suggestions from the assessment participants.

### Ethical Considerations

This research is dispensed of the ethical committee's approval, the User Feedback for Continuous Improvement being a normal educational practice and classroom management method conducted in educational settings. Specifically, as non-interventional research dealing with practical habits analysis the Rouen University Hospital ethical committee does not ask for submitting such kind of research to the ethical committee. Moreover, the whole project SaNuRN that comprises CIDHR has been approved as a whole by the Delegation of Digital Health of the French Ministry of Health and the French National Research Agency [38].

## Results

### Resource Discovery and Indexation in CIDHR

The outcomes of the CIDHR resource identification and evaluation processes were executed by a dedicated team of 3 librarians from the URN—Rouen University Hospital DDH, aiming to collect and curate an extensive collection of digital health teaching and learning resources. Our identification strategies yielded a diverse and expansive pool of digital health educational resources through diligent exploratory searching of academic websites and platforms (eg, a systematic review of French universities’ digital health departments and several French national agencies such as *Agence Nationale de sécurité du médicament* and *La Haute Autorité de Santé*) [26,27]. We successfully identified a continuously updating substantial number of resources catering to various aspects of digital health education. The use of search engine alerts (eg, Google Alerts [39] and PubMed alerts [40]), social media monitoring (eg, LinkedIn [41]), newsletters, and professional network notifications (of posts in groups of interests) also contributed significantly to the resource identification process.

In the last year, we identified approximately 500 valuable resources. It is noteworthy that the identified resources reflect a multilingual character (in particular, English). However, to align with the SaNuRN grant requirements, a substantial proportion (>90%) of the resources is in French. However, we ensured a representation of diverse languages to accommodate a wide-ranging audience interested in digital health education. In addition, we supported the ongoing internationalization and English-drafted teaching and self-learning introduced in the French higher education curricula.

### Resources Evaluation

The “resource evaluation process” disclosed in the *Methods* section together with its 3 fundamental criteria ensures that each resource included up to now has been evaluated for relevance, “accuracy and currency,” and accessibility.

The relevance was scrutinized to ascertain its suitability for teaching and learning digital health education to serve the needs of both students and lecturers. As a result, a significant portion of the identified resources clearly aligned with digital health education objectives (323/503, 64.2%). The 35.8% (180/503) of resources that were excluded were in the scope of digital health, but they did not sufficiently focus on real teaching resources.

Furthermore, each one of the remaining resources was subjected to a rigorous assessment of “accuracy and currency” to ensure its alignment with up-to-date research findings and adherence to best practices within the digital health field. The evaluation step revealed that some resources did not meet these accuracy and currency criteria and were rejected (approximately 36%).

The “accessibility” of the educational supports is a critical aspect emphasized in CIDHR resource evaluation to include in the catalog materials that can effectively be used by a broad range of the digital health educational community, including individuals with disabilities. This evaluation highlighted the commitment of many resources to accessibility.

If a potential resource does meet any one of these criteria, it does not move to inclusion in CIDHR and remains in a secondary list of resources to be periodically re-evaluated for future inclusion.

Resources that successfully passed all 3 evaluation criteria were promptly included in CIDHR. These resources are diligently indexed and tagged as described in the *Methods* section.

### Tailored Learning Paths: Metadata, Training Matrix, and Resource Cataloging in CIDRH

The semantic search engine of CIDHR based on MIMO and FNRDH allows user-friendly access to previously indexed and tagged resources. At the end of September 2023, CIDHR comprised 371 available resources in the digital health field relevant to students and teaching staff from the first academic year of academic studies to lifelong continuing education. The French grant required that 80% of the effort should focus on the bachelor “degree level.” Therefore, approximately all the 371 resources included in CIDHR are focusing on bachelor’s students.

CIDHR is constantly expanding, with plans to incorporate increasingly as much as possible digital health teaching resources from the French health–related studies curricula over the next few years [6].

**Figure 3:**
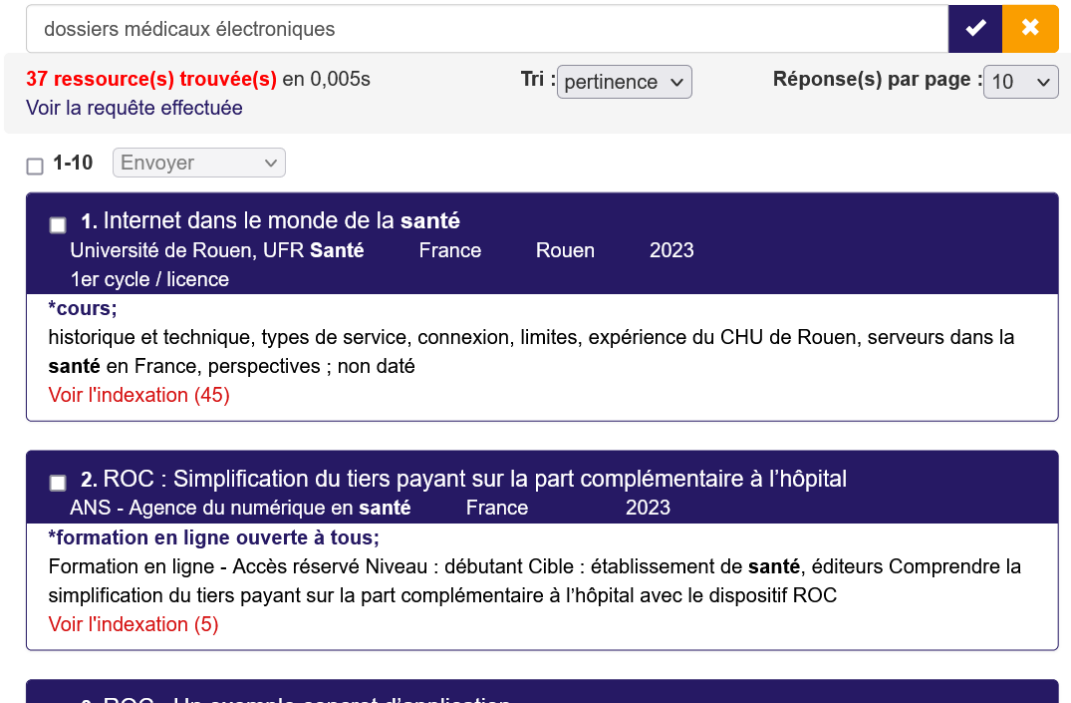
Example of results to the query “dossiers médicaux électroniques” (in English, “electronic health records” or EHRs). CIDHR: Catalog and Index of Digital Health Teaching Resources; CISMeF: Catalogue et Index des Sites Médicaux en langue Française (Catalog and Index of Medical Sites in French Language).

Figure 3 shows an example of the results for the query “dossiers médicaux électroniques” (in English, “electronic health records” or EHRs).

**Figure 4:**
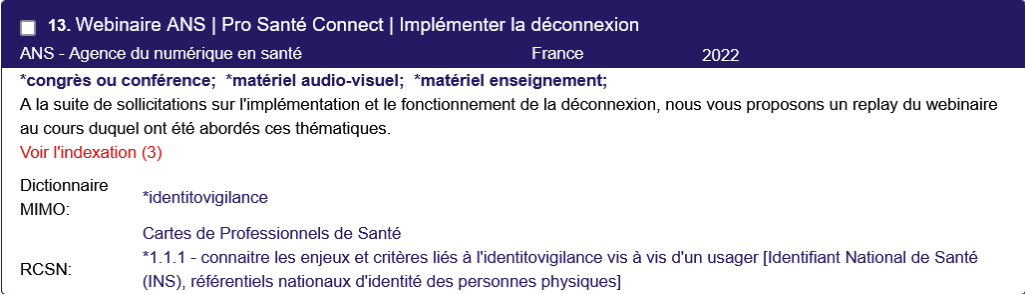
Example of an indexed resource in Catalog and Index of Digital Health Teaching Resources (CIDHR) comprising the following metadata: resource title, resource published and author, country, year of publication, type of document, an abstract, and a list of the terms and concepts used for indexation (here with both Medical Informatics Multilingual Ontology [MIMO] and French National Referential on Digital Health [FNRDH]).

Figure 4 shows an example of a digital health educational resource, as a bibliography card, indexed using MIMO and FNRDH, which is an example of CIDHR’s capabilities. A CIDHR bibliographic card comprises the following metadata: (1) the resource title, (2) the resource publisher or author, (3) the country of the source, (4) the year of publication, (5) the type of resource, (6) an abstract presenting the resource, and (7) a list of the terms and concepts used to index the resource with regard to controlled vocabularies and referential such as MIMO and FNRDH (Figure 4).

The resource is written in French and focuses on EHRs, a concept defined in both MIMO (ie, “dossiers médicaux électroniques”) and FNRDH (ie, “Interagir de manière adaptée entre professionnels, avec l’usager, les aidants et accompagnants et avec les institutions et administrations,” in English, “Interact in an appropriate manner between professionals, with the healthcare customer, caregivers and companions and with institutions and administrations”; and “Utiliser les outils et services socles adaptés et identifier leur articulation avec d’autres dossiers partagés,” in English, “Use the appropriate basic tools and services and identify their connection with other shared files”). It educates the learners on the fundamentals and the importance of the EHRs, making it an invaluable resource for anyone looking to enhance their digital health knowledge.

To facilitate the indexing process with FNRDH, which presents considerable complexity for medical librarians, the SaNuRN pedagogical team has established manual associations between MIMO and FNRDH concepts. For instance, this involves manually linking the MIMO concept with the FNRDH competency. It is essential to clarify that this mapping relation does not constitute a strict “exact match”; instead, it means that when a librarian indexes a teaching resource using a MIMO concept (eg, “electronic medical records”) associated with an FNRDH ability (eg, “Interact appropriately between professionals, with the healthcare customer, caregivers and companions and with institutions and administrations”), the educational resource is also indexed with this corresponding FNRDH competency.

Nevertheless, certain cases require manual indexing with FNRDH by medical librarians, primarily because of the absence of the MIMO concepts for specific capacities, still not defined and implemented in MIMO, such as the “lifecycle of health data.” Thus, to minimize the dependency on manual FNRDH indexing, the SaNuRN pedagogical team is actively developing MIMO concepts and establishing mappings between MIMO and FNRDH concepts, including those pertaining to the lifecycle of health data.

In addition, as a part of CIDHR capacities, the end-user process for any query to deliver an organized list of educational resources is considered. The first item on the list must be studied first, followed by the second item, and so on. This organized list is manually created for each FNRDH competency; in other words, we create a breadcrumb navigation for teaching and learning resources linked to each FNRDH competency. Currently, this organized list is familiar to all the students in all the fields of study. In the future, this organized list will be, when relevant, adapted to fit with the requirements of each field of study (eg, medicine, nursing), degree level (eg, bachelor, residency), and targeted level competencies or skills (eg, beginner, intermediate, and advanced).

### User Feedback for Continuous Improvement

To assess CIDHR’s usability and acceptance among users, we collected informal feedback from a select group comprising both first-year health students (10/15, 67%) and teaching staff (5/15, 33%). Their feedback universally reflected a positive sentiment, characterizing the platform as remarkably user-friendly and easily navigable. Moreover, they lauded the platform’s existing resource collection, founded on rigorous content quality control, and appreciated its ongoing expansion. Notably, users articulated their assessment, highlighting CIDHR’s comprehensiveness, precision, and user-friendliness. Nonetheless, their constructive suggestions included the need for augmenting multilingual resources and offering more comprehensive resource information, particularly with respect to metadata. In the users’ collective perception, CIDHR was deemed a one-stop destination for discovering high-quality digital health resources. An additional commendable attribute was the platform’s unrestricted accessibility, which rendered it a valuable asset for all users.

Moreover, additional suggestions related to the need for more multilingual resources and comprehensive metadata were noted (eg, field of study, resource language, and resource scoring; Table 1).

**Table 1:** Summary of the feedback collected during the Catalog and Index of Digital Health Teaching Resources user experience informal assessment.

Feedback category	Students	Lecturers
Usability	User-friendliness and “easily navigable” capabilities	User-friendly and “simple to understand”
Content quality	Valuable, “easy to understand”	Valuable, comprehensiveness
One-stop shop potential	Free resources, easy to access, on various relevant content	Real one-stop shop freely and unrestricted accessible, especially available digital health resources in their academic (ie, learning, teaching, and research) and professional activities
Participants suggestions for improvements	More than French-only resources, in particular English, but also Arabic, Spanish and Portuguese (native language of the students)	More metadata on bibliographic card; more than French-only resources, in particular English

## Discussion

### Overview

The integration of digital technologies in health care and medical education is becoming increasingly vital. This study introduces CIDHR as part of the SaNuRN project to enhance digital health education in France. CIDHR is a comprehensive digital platform that indexes and organizes educational resources related to digital health, catering to students and health care professionals. This discussion explores the strengths and limitations of CIDHR, potential future perspectives, and the impact on digital health education.

### Strengths and Limitations

CIDHR is the heart of a digital health educational platform that provides an extensive array of inclusive and accessible teaching and learning resources to a diverse global audience in the health care professional education landscape. CIDHR has a large and continuously expanding collection of up-to-date and relevant digital health resources that serves as a one-stop shop related to all aspects of digital health education needs, catering to lecturers, students, and professionals alike.

CIDHR is committed to providing comprehensive support to French-speaking individuals seeking digital health education. To ensure that language barriers do not impede access to educational resources, CIDHR has indexed a wide range of materials in multiple languages, in addition to its French language resources. These materials are designed to cater to diverse linguistic needs and are available to all individuals seeking to enhance their digital health knowledge. With CIDHR’s vast collection of indexed educational resources, individuals can access high-quality information and support regardless of their native or daily spoken language.

To improve resource indexing and search precision, CIDHR uses controlled vocabularies such as MIMO and FNRDH, which enable users to locate relevant educational materials that align with their specific digital health skills and competencies with ease. Moreover, CIDHR prioritizes resource accessibility, making its platform suitable for a broad audience, including individuals with disabilities [42,43]. Thus, CIDHR, being based on a multilingual semantic search engine, would enhance accessibility and inclusivity. By looking at all (even mainly French speakers currently) health care professionals, researchers, and students, CIDHR allows them to have access to a broader range of educational resources, fostering a more inclusive learning environment. This inclusivity aligns with the principles of health equity and diversity in medical education [44]. Furthermore, the CIDHR platform’s user-friendly interface and straightforward navigation enable users to connect with relevant educational resources quickly and efficiently.

By looking at these advantages and the SaNuRN aim to facilitate digital health educational resources, the current corpus, including 371 elements, will be expanded by continuing the collection and evaluation process, in parallel with cooperation with as many possible faculties and schools of health (ie, 31 medical schools in France). We expect approximately 700 CIDHR resources by mid-2024.

However, some limitations have been identified. First, although CIDHR supports mainly French resources, it would benefit from expanding its multilingual and international support to make it more accessible to a global audience of the digital health education community. Second, it is necessary to expand CIDHR resource collection to incorporate more digital health resources from diverse sources allowing providing them to the educational community and industry insights. Third, although SaNuRN plans to provide personalized learning paths to users, via CIDHR, it is crucial to ensure that these paths are effective and tailored to the individual needs of each user, which requires further research and development [45,46]. Fourth, integrating CIDHR with the learning management systems used by educational institutions would streamline access to digital health resources for students and educators. However, it is crucial to ensure that the integration is smooth and that CIDHR is easy to use within these systems. Finally, developing a feedback and rating system for resources would be helpful in enabling users to identify the most valuable and reliable materials within the platform. However, it is vital to design the system carefully to ensure that it is fair and unbiased. Moreover, it is important to note that CIDHR is under development, and there may be some bugs or glitches in the system. In addition, some features may not be fully implemented.

### Future Perspectives

Handling the current limitations of CIDHR opens a wide range of perspectives.

To improve the accessibility and user-friendliness of CIDHR, the SaNuRN team will look at different paths. First, expanding multilingual support to cater to a wider global audience (over the French-speaking community) by indexing (based on MIMO as a multilingual ontology dedicated to digital health) more resources in more languages MIMO on the platform. In addition, CIDHR enrichment will benefit from the SaNuRN team’s international partnerships and collaborations to expand CIDHR resource collection and promote knowledge exchange to enrich the user experience [23,47]. Moreover, an additional enhancement is planned to provide personalized learning paths to users based on their profiles, such as their field of study, degree level, CIDHR personal and similar user use, to enable tailored educational experiences and effectiveness. Furthermore, CIDHR will be integrated with the learning management systems used by educational institutions to streamline access to digital health resources for students and educators (eg, Moodle [48]). Finally, CIDHR will benefit from the development of a feedback and rating system for resources not only to help users identify the most valuable and reliable materials within the platform but also to allow the SaNuRN team project to get feedback on the resource collection, indexing, and tagging processes from mass users’ practice. All these measures will augment CIDHR utility and enrich the user experience.

### Conclusions

CIDHR represents a significant advancement in digital health education, offering a diverse, accessible, and validated resource collection. Although it has strengths in its multilingual approach, controlled vocabularies, and user-friendliness, addressing resource evaluation challenges and enhancing resource information are areas for continuous improvement. The future perspectives for CIDHR include further expansion, collaboration, personalized learning, integration, and user feedback mechanisms, all aimed at enriching the digital health education experience for students and health care professionals.

To the best of our knowledge, no prior published research has described a multilingual semantic search engine to query a digital health educational repository to be used by any health-related field student and lecturer. This is also because of the uniqueness of the development of the Health Terminology/Ontology Portal and MIMO by the members of the SaNuRN team. These projects have no equivalent to date.

The hypothesis that guided this part of the SaNuRN research and that we have validated is that controlled vocabularies and knowledge and skills referential dedicated to medical informatics and digital health, such as MIMO [22,23] and FNRDH [24], to index related educational resources, are effectively increasing the availability and accessibility of these resources to the health care–related community. This approach is possible as MIMO and CIDHR search engine are multilingual.

A European project called the HosmartAI (Hospital Smart development based on AI) project deals with the digital transformation of the European health care sector to make the European health care system more strong, efficient, sustainable, and resilient. CIDHR can play an important role in acquisition of literacy in digital health for professionals [49]. The European Federation for Medical Informatics is taking part in different projects such as HosmartAI and as a collaboration and cooperation-oriented scientific and academic international organization, it can help disseminate information about CIDHR to promote its use by an increasing number of members of the digital health educational community worldwide.

However, the need to develop and improve digital health competencies for medical learners and broadly for health-related students and professionals is an established objective worldwide [45,50,51]. As a fact, prior studies evaluating digital health competencies among German medical students have shown a significant improvement after a digital health teaching course was introduced in their curriculum, although most students found that digital health is not sufficiently taught in undergraduate medical education, while it may influence everyday work of physicians [52].

Thus, CIDHR will have an important role on the educational grounds to improve digital health literacy of students and lecturers and to increase their engagement with these ubiquitous ways of delivering and receiving health care [46,53].

CIDHR is a fair and findability, accessibility, interoperability, and reusability principles–focused platform looking at making “findable” educational resources by using a one-stop-shop portal approach, “accessible” by integrating these resources available overtime and by anyone (ie, including people with disabilities), “interoperable” by making these resources readable in the most common formats (PDF files and video and audio support on browser-embedded readers, such as YouTube), and finally “reusable” by providing resources freely distributed and under open access licensing [54-56].

## Abbreviations

CAU: Côte d’Azur University
CIDHR: Catalog and Index of Digital Health Teaching Resources
CISMeF: Catalogue et Index des Sites Médicaux en langue Française (Catalog and Index of Medical Sites in French Language)
DCMI-MT: Dublin Core Metadata Terms
DDH: department of digital health
EHR: electronic health record
FNRDH: French National Referential on Digital Health
HosmartAI: Hospital Smart development based on AI
KOS: knowledge organization system
LOM: Learning Object Metadata
MeSH: Medical Subject Headings
MIMO: Medical Informatics Multilingual Ontology
SaNuRN: Santé Numérique Rouen Nice
SoM: sets of metadata
URN: University of Rouen Normandy

## Data Availability

The data sets generated during or analyzed during this study are available from the corresponding author upon reasonable request.
